# A contribuição da pluralidade de modelos na pesquisa do sofrimento pós-traumático

**DOI:** 10.1590/0102-311XPT205524

**Published:** 2025-01-27

**Authors:** Deyvianne Thaynara de Lima Reis

**Affiliations:** 1 Universidade do Estado do Pará, Belém, Brasil.

Prezadas Editoras,

Li com grande interesse o ensaio *A Soberania do Visível: Como a Memória Traumática se Torna Estresse Traumático* ^1^, e considero-o significativo no campo do transtorno do estresse pós-traumático (TEPT). Os autores, no decorrer do tempo, do século XIX ao XX, relataram o impasse entre a memória literalizada do trauma e a memória pulverizada do trauma, logo, o trauma passou de um conceito cognitivo de memória para um problema de pesquisa neurocientífica do estresse, assim as concepções abstratas da memória deram lugar ao vocabulário materialista do estresse.

Em relação à narrativa histórica relatada no ensaio, gostaria de fazer a seguinte contribuição: a virada neurocientífica está em grande expansão e foi eleita com destaque pelo governo dos Estados Unidos como prioritária na década de 1990, pois ficou conhecida como a “Década do Cérebro”. Muitos também consideram o século XXI o século do cérebro, no qual as grandes conquistas da humanidade estarão dirigidas para a compreensão das funções neurais humanas ^2^. No entanto, submeter a psicanálise à neurociência como se houvesse uma hierarquia entre elas, simplesmente importando o vocabulário da segunda para a primeira, soa equivocado, é necessário fugir dos reducionismos. A possibilidade de dialogar com outros campos que tratem do funcionamento mental pode ser enriquecedora do ponto de vista da pesquisa e da clínica, desde que guardando as particularidades de cada disciplina ^3^.

Em uma análise crítica, cabe destacar que tanto a análise subjetiva, quanto a objetiva são pertinentes no estudo do TEPT, pois existe um leque de ferramentas terapêuticas que podem auxiliar as vítimas dessa patologia, como a técnica comportamental cognitiva, a psicanálise e a abordagem farmacológica, cada uma com suas diferenças e abordagens distintas contribuem significativamente para o entendimento do funcionamento da mente e do estresse. O postulado de sensibilização neurofisiológica, pedra angular para uma abordagem neurobiológica dos transtornos do espectro traumático, junto com a pesquisa de condicionamento clássico, teorias do estresse e hipótese evolucionárias só confirmam a gama de teorias que podem embasar o TEPT, com visões divergentes, porém pertinentes, com atuação em conjunto para a evolução de vítimas do TEPT ^4^.

Adentrando mais especificamente na memória, ela nem sempre é uma lembrança real dos acontecimentos, o traumatizado vive mais refém das impressões do que do acontecimento exato ocorrido ^4^. Ele vive em função de uma impressão vívida, modulada pela emoção do momento traumatizante, ficando estagnado na ruptura psíquica da emoção com a percepção ^5^. Além disso, algumas funções da memória podem ficar prejudicadas depois de um TEPT. A exposição a eventos traumáticos é o aspecto de contexto mais investigado atualmente, indicando associações positivas com a doença. Essa exposição ao evento é caracterizada por pensamentos intrusivos, pesadelos e *flashbacks*, esquiva de lembrança do trauma, cognições negativas, hipervigilância e distúrbios do sono ^6^. O problema do trauma é tanto uma questão de quantidade, quanto de qualidade, pois a concretude da neurobiologia não pode deixar de lado a especulação de teorias subjetivas, psíquicas, comportamentais e evolucionárias.
